# Prognostic and predictive value of common mutations for treatment response and survival in patients with metastatic colorectal cancer

**DOI:** 10.1038/sj.bjc.6605164

**Published:** 2009-07-14

**Authors:** J Souglakos, J Philips, R Wang, S Marwah, M Silver, M Tzardi, J Silver, S Ogino, S Hooshmand, E Kwak, E Freed, J A Meyerhardt, Z Saridaki, V Georgoulias, D Finkelstein, C S Fuchs, M H Kulke, R A Shivdasani

**Affiliations:** 1 Department of Medical Oncology, University Hospital, Heraklion, Crete, Greece; 2 Laboratory of Tumor Biology, University of Crete, Heraklion, Crete, Greece; 3 Department of Medical Oncology, Dana-Farber Cancer Institute, Boston, Massachusetts, USA; 4 Department of Biostatistics, Massachusetts General Hospital, Boston, Massachusetts, USA; 5 Department of Pathology, Laboratory of Pathology, University Hospital, Heraklion, Crete, Greece; 6 Department of Pathology, Brigham and Women's Hospital, Boston, Massachusetts, USA; 7 Department of Medicine, Massachusetts General Hospital, Boston, Massachusetts, USA; 8 Department of Medicine, Harvard Medical School, Boston, Massachusetts, USA

**Keywords:** KRAS, PIK3CA, BRAF, mutation, prediction, metastatic CRC

## Abstract

**Background:**

We address the prognostic and predictive value of *KRAS*, *PIK3CA* and *BRAF* mutations for clinical outcomes in response to active agents in the treatment of metastatic colorectal cancer (mCRC).

**Methods:**

We determined *KRAS*, *BRAF* and *PIK3CA* mutations in tumours from 168 patients treated for mCRC at two institutions. All patients received 5-FU-based first-line chemotherapy and treatment outcome was analysed retrospectively.

**Results:**

*KRAS*, *BRAF* and *PIK3CA* mutations were present in 62 (37%), 13 (8%) and 26 (15%) cases, respectively. Multivariate analysis uncovered *BRAF* mutation as an independent prognostic factor for decreased survival (hazard ratio (HR) 4.0, 95% confidence interval (CI) 2.1–7.6). In addition, patients with *BRAF*-mutant tumours had significantly lower progression-free survival (PFS: HR 4.0, 95% CI 2.2–7.4) than those whose tumors that carried wild-type *BRAF*. Among 92 patients treated using chemotherapy and cetuximab as salvage therapy, *KRAS* mutation was associated with lack of response (*P*=0.002) and shorter PFS (*P*=0.09). *BRAF* (*P*=0.0005) and *PIK3CA* (*P*=0.01) mutations also predicted reduced PFS in response to cetuximab salvage therapy.

**Conclusions:**

These results underscore the potential of mutational profiling to identify CRCs with different natural histories or treatment responses. The adverse significance of *BRAF* mutation should inform patient selection and stratification in clinical trials.

Combinatorial and sequential administration of 5-fluorouracil (5-FU), oxaliplatin and irinotecan has substantially increased the overall survival (OS) in metastatic colorectal cancer (mCRC), although treatment goals remain primarily palliative (Grothey *et al*, 2004). Monoclonal antibodies (mAb) bevacizumab, against the vascular endothelial growth factor (VEGF) receptor, and cetuximab or panitumumab, against the epidermal growth factor receptor (EGFR), expand treatment options and further improve progression-free (PFS) and OS (Cunningham *et al*, 2004; Hurwitz *et al*, 2004; Van *et al*, 2007).

The genetic underpinnings of colorectal cancer (CRC) are especially well characterized (Vogelstein and Kinzler, 2004) and include common somatic mutations in the *APC*, *TP53* and *KRAS* genes, followed in frequency by *PIK3CA* and *BRAF* mutations (Wood *et al*, 2007). Activating mutations in the *KRAS*, *BRAF* and *PIK3CA* oncogenes deregulate growth-factor pathways, stimulate cell proliferation and promote metastasis (Huang *et al*, 2007; Schubbert *et al*, 2007). Presence of *KRAS* mutations in primary CRC portends a poor response to monotherapy and combination therapy using anti-EGFR mAB cetuximab or panitumumab (Amado *et al*, 2008; Karapetis *et al*, 2008; Lievre *et al*, 2008). In one study that included few patients with stage IV disease and *BRAF* mutations, the latter were associated with limited survival in patients with microsatellite-stable (MSS) disease (Samowitz *et al*, 2005); mutations in exons 9 or 20 of the *PIK3CA* gene are associated with lower relapse-free survival in patients with stage II or III CRC (Kato *et al*, 2007). These data notwithstanding, there is limited understanding of the extent to which common *KRAS*, *BRAF* and *PIK3CA* mutations influence clinical outcomes in patients with mCRC treated with chemotherapy and/or biological therapy. Treatments are selected largely without regard for tumour mutation profiles, although recent evidence linking *KRAS* mutation with poor response to EGFR mAb (Amado *et al*, 2008; Karapetis *et al*, 2008; Lievre *et al*, 2008) now influences decisions in the clinic.

We postulated that mutations in some of these oncogenes would correlate with overall prognosis and with treatment outcomes in patients receiving different regimens. To test these hypotheses, we first evaluated the prognostic significance of *KRAS*, *BRAF* and *PIK3CA* mutations in a cohort of patients with mCRC, treated using 5FU-based chemotherapy. Using a uniform catalogue of retrospective but detailed clinical information, we then tested the predictive value of these mutations on patient outcomes after treatment using the most common therapeutic regimens.

## Materials and methods

### Patients and study design

Tumour samples were collected from patients with histologically confirmed mCRC, treated at two centers, Dana-Farber/Partners Cancer Care (Boston, MA, USA) and University Hospital of Heraklion (Heraklion, Crete, Greece), between January 2004 and March 2007. All patients who gave informed consent and whose colectomy samples were available for molecular analysis were included in the analysis, without further selection. Failure to participate in the tumour registry was unusual, and the cohort essentially represents consecutive patients with mCRC who were treated at the two academic centres and gave informed consent for molecular analysis of DNA from the primary tumour. Institutional ethics committees approved the study.

First-line and salvage regimens, selected at the discretion of the treating physician, followed common practices in mCRC (summarized in Table 1). Patients were evaluated at baseline and after every four cycles of therapy, with occasional variation, as clinically indicated, during the treatment. Disease status was coded, without the knowledge of the mutational profile, after retrospective review of physician notes and reports of staging radiographs, including tumour measurements; efficacy was assessed on an intent-to-treat basis. Disease progression was defined as the appearance of any new lesion or a greater than 20% increase in the sum of the longest diameter (LD) of target lesions, extracted from radiology reports, and taking as reference the lowest value recorded in the treatment period. Response toward cetuximab was defined as the reduction, by at least 30%, in the sum of the LD of target lesions compared with the baseline sum.

### Specimen Characteristics and Assay Methods

Formalin-fixed, paraffin-embedded tumour sections were reviewed by pathologists to confirm the diagnosis and define tumour-enriched areas for dissection. From each paraffin block of representative tumour areas, we stained 10 serial sections of 8 *μ*m thickness with nuclear fast red (Sigma-Aldrich, St Louis, MO, USA) and isolated malignant cells by scrape dissection under a binocular microscope. Macrodissected cells were lysed in buffer containing Proteinase K (Puregene, Gentra Systems, Minneapolis, MN, USA) at 60 °C for 72 h, followed by DNA extraction according to the manufacturer's protocol.

Mutations were detected in the Dana-Farber sample set by Sequenom mass-spectrometric genotyping (Thomas *et al*, 2007) after whole-genome amplification by PCR, and in the Heraklion samples by Sanger sequencing after PCR amplification of KRAS exon 2, *BRAF* exon 15, and *PIK3CA* exons 9 and 20. PCR conditions and the primers used to amplify specific exons are listed in Supplementary Table 1. Results from the two different methods for detecting mutations were cross-confirmed on the other platform, along with positive and negative controls.

### Statistical analysis

Associations between treatment response and mutation status or baseline characteristics were assessed using the Fisher's exact test for dichotomous variables, or logistic regression for continuous variables. PFS was measured from the last radiographic study before the initiation of first-line therapy to the first radiographic documentation of disease progression or death, and OS was calculated from the date of diagnosis of metastatic disease to death due to any cause. For cetuximab treatment, PFS was calculated from the date of documented progression after the previous treatment to the next documentation of disease progression or death. Kaplan–Meier curves were used to describe the proportion of patients who remained free of events over the follow-up period. Associations between prognostic factors and PFS or OS were examined using Cox proportional hazards regression models; we report hazard ratio (HR) estimates and their 95% confidence intervals (CI).

We used Cox regression models with interaction terms to assess whether mutational effects varied across treatment subgroups. For each mutation, three hypotheses were tested: (1) whether effect on first-line PFS varied according to regimen (oxaliplatin based, irinotecan based or both); (2) whether the effect on first-line PFS varied according to inclusion or exclusion of bevacizumab; and (3) whether the effect on PFS for cetuximab varied according to the line of treatment (first *vs* ⩾third). All reported *P*-values are two-sided and not adjusted for multiple testing.

## Results

### Patient and tumour characteristics, treatments and clinical outcomes

We determined with confidence the mutational status for *KRAS* exon 2, *BRAF* exon 15, and *PIK3CA* exons 9 and 20 in 168 consecutive patients with mCRC, for whom a representative sample from the primary tumour was available for molecular analysis of DNA. Disease characteristics were typical for mCRC in North America and Europe (Table 2), and patients were treated according to prevailing practice patterns (Table 1). *KRAS* mutations were detected in 62 (37%), *BRAF* mutations in 13 (8%) and *PIK3CA* mutations in 26 (15%, 18 in exon 9 and 8 in exon 20) primary tumours, respectively. Seven tumours carried both *KRAS* and *PIK3CA* mutations, whereas *BRAF* mutations did not co-occur with the others; these frequencies closely match those in published reports (Wood *et al*, 2007) and were similar between the US and Greek cohorts (Supplementary Table 2). Mutations in any gene did not correlate significantly with patient gender or age, stage at diagnosis, or histological grade (all *P*-values >0.05). As patient characteristics, mutation frequencies and treatment regimens were all typical for CRC, the results of our analysis should serve as a useful guide for clinical practice.

The median time from initial diagnosis to diagnosis of metastatic disease was 18.4 months (95% CI 14.4–20.4) for patients with stage I–III disease at presentation and 0.6 months (95% CI 0.1–1.4) for patients classified with stage IV disease. The median time from the last radiological assessment to the initiation of first-line chemotherapy was 0.6 months (95% CI 0.4–0.8), whereas median duration from the diagnosis of metastatic disease was 1.2 months (95% CI 0.9–1.3).

**Figure 1:**
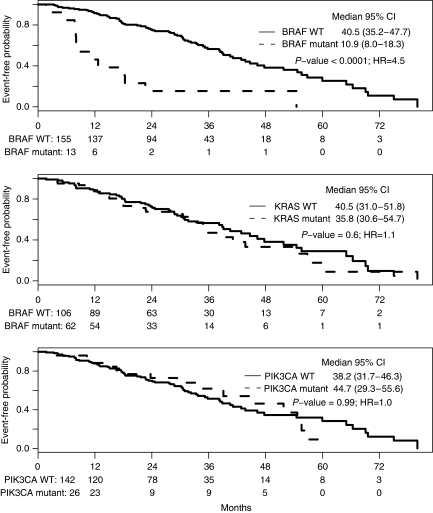
Overall survival after the diagnosis of metastatic disease, analysed by mutation status.

OS was similar between the two participating centres (median 38.9 months, 95% CI 33.4–46.3 months) and higher than other published values (Grothey *et al*, 2004; Hurwitz *et al*, 2004). Considering that patients were not selected on the basis of performance status or other clinical criteria, one possibility for improved outcomes may be that after initial response to systemic treatment, 30 out of 50 patients with solitary metastases (representing 18% of the total cohort) had metastases resected. Response to particular agents was, however, monitored in all patients and we examined the role of metastatectomy as a variable. Univariate analysis (Table 2) showed significant associations of OS with age >65 years (*P*=0.03), undifferentiated (grade 3) primary tumours (*P*=0.02), colonic *vs* rectal location (*P*=0.02), solitary metastasis (*P*=0.008) and *BRAF* mutations (*P*<0.0001, Figure 1). When all factors significant in univariate analysis were subjected to multivariate analysis (Table 3), only *BRAF* mutation remained as an independent prognostic factor for reduced OS (HR 4.0, 95% CI 2.1–8.0).

### Impact of mutation status on the outcome of first-line chemotherapy

Combination chemotherapy was administered in the first line in 161 patients (96%), with or without mAb supplementation (Table 1); 5 patients (3%) received 5-FU as the only cytotoxic agent. One hundred patients (60%) were treated with the folinic acid–fluororuracil–oxaliplatin (FOLFOX) regimen, with or without the addition of a mAb, in the first line and 97 patients (58%) received bevacizumab in addition to fluoropyrimidine-based chemotherapy; a smaller fraction, 24% (44 patients) was treated with irinotecan in the first line. Seventeen patients (10%) received both oxaliplatin and irinotecan (Table 1). In all, 106 patients (63%) received all three active chemotherapy drugs and 87 patients (52%) received all five active agents at some point in their disease course.

**Figure 2:**
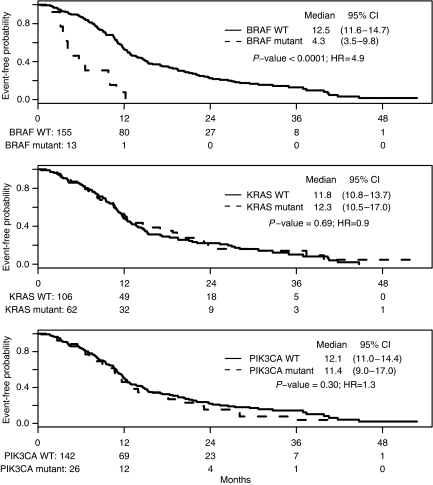
Progression-free survival after first-line chemotherapy, analysed by mutation status.

*KRAS* or *PIK3CA* mutations did not influence PFS after first-line therapy (Figure 2). In contrast, patients with *BRAF*-mutant tumours had significantly lower PFS (median 4.3 *vs* 12.5 months; *P*<0.0001) compared with patients whose primary tumours carried only wild-type (WT) *BRAF* alleles (Figure 2). The association between *BRAF* mutation and reduced PFS (HR 4.9, 95% CI 2.7–9.0) after first-line therapy (Table 3) was similar for patients receiving oxaliplatin-, irinotecan- or both (interaction test *P*=0.7), and similar for patients receiving a regimen with or without bevacizumab (interaction test *P*=0.8). Specifically, *BRAF* mutation predicted more rapid disease progression in patients treated with first-line oxaliplatin- (HR 6.4, 95% CI 2.6–15.6), irinotecan- (HR 4.1, 95% CI 1.5–11.3), or oxaliplatin and irinotecan (HR 7.9, 95% CI 1.3–48.2), as well as bevacizumab-containing (HR 5.1, 95% CI 2.4–11.1) regimens (Supplementary Table 3). Thus, *KRAS* and *PIK3CA* mutations did not show predictive value with respect to cytotoxic agents and our observations single out *BRAF* mutation in the primary tumour as an adverse factor, independent of the first-line treatment.

### Impact of mutation status on the outcome of salvage cetuximab therapy

**Figure 3:**
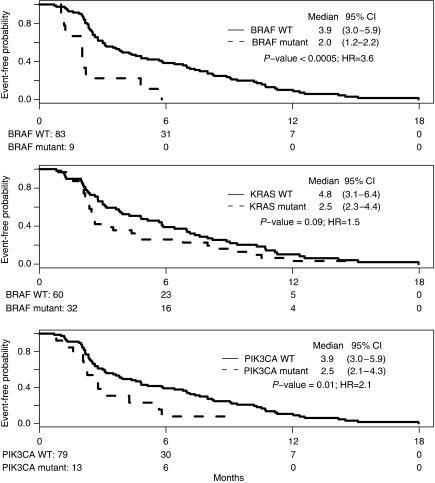
Progression-free survival after salvage cetuximab therapy in 92 patients, analysed by mutation status.

One hundred patients were treated with cetuximab, 8 in the first line, 37 in the second and 55 in the third or higher, always in combination with chemotherapy. All patients receiving cetuximab in the second or subsequent line were refractory to previous regimens. Restricting analysis to the 92 patients who received cetuximab combinations as salvage therapy, none of the 32 patients with a *KRAS*-mutant tumour responded to treatment, whereas objective responses occurred in 14 of 60 (23%) patients whose tumors harboured only WT *KRAS* alleles (*P*=0.002). This striking difference confirms the results from prospective trials of cetuximab or panitumumab monotherapy (Amado *et al*, 2008; Karapetis *et al*, 2008; Lievre *et al*, 2008) and validates our approach to identify associations to guide the treatment of mCRC. It should be noted that none of the 9 patients with a *BRAF*-mutant tumour responded to cetuximab, whereas objective responses occurred in 14 of 83 (17%) patients whose tumours carried WT *BRAF* alleles. Presence of *PIK3CA* mutations showed no correlation with objective tumour responses to cetuximab. PFS was significantly lower among patients whose tumours carried mutations in *BRAF* (2.0 *vs* 3.9 months, HR 3.6, 95% CI 1.8–7.4), *PIK3CA* (2.5 *vs* 3.9 months, HR 2.1, 95% CI 1.2–3.9) or any of the three genes (2.5 *vs* 6.4 months, HR 2.1, 95% CI 1.3–3.2), and marginally lower in patients with *KRAS*-mutant tumours (2.5 *vs* 4.8 months, HR 1.5, 95% CI 0.9–2.3), compared with patients whose primary tumors were wild type at each locus (Figure 3, Supplementary Table 3). Importantly, patients whose tumour had *BRAF* or *PIK3CA* mutation had shorter PFS regardless of whether cetuximab was administered in the second, or third and higher lines (interaction tests *P*=0.5 and 0.6, respectively). Thus, the lack of EGFR mAb response observed in the setting of mutant *KRAS* (Schubbert *et al*, 2007; Amado *et al*, 2008) extends to other common mutations that deregulate cellular signalling pathways, especially *BRAF* and probably even *PIK3CA*. Lower PFS did not translate into differences in OS between patients with WT or mutant *KRAS* and *PIK3CA* alleles in the primary tumour.

## Discussion

Among the many genetic alterations in CRC, particular point mutations in three oncogenes, *KRAS*, *PIK3CA* and *BRAF*, occur at sufficiently high frequencies to be implicated in disease pathogenesis and as targets for molecular therapy (Wood *et al*, 2007). Each of these targets is the subject of intense drug development efforts. Appreciation of the predictive significance of underlying oncogene mutations will aid in design of clinical trials of new agents, administered either alone or in combination with other drugs, and in interpreting results from new and old trials. Moreover, current treatment approaches toward mCRC rarely consider biological properties associated with individual mutations, mainly because there is a paucity of information about the influence of specific mutations on natural history or the response to particular treatments. We report the results of retrospective investigation designed to help define the role of tumour mutation profiles in the management of patients with mCRC. Even though treatment choices and staging studies were not dictated by a strict clinical trial, the results are robust and relate well to current treatment paradigms.

A principal finding was that *BRAF* mutation the primary tumour marks patients who carry an especially poor prognosis, regardless of specific treatment regimen. Compared with patients with *BRAF*^WT^ tumours, those with *BRAF*^*MUT*^ tumours have significantly higher likelihood of disease progression (*P*<0.0001) or death (*P*<0.0001) with any current regimen (Table 3); the *BRAF* V600E mutation predicted independently for early relapse on first-line therapy (HR 4.0, 95% CI 2.2–7.4) and death (HR 4.0, 95% CI 2.1–8.0). These data warrant confirmation and should provoke thoughtful consideration of the risks and benefits of administering 5-FU, oxaliplatin or irinotecan in such patients, who should probably also be considered as a distinct group by stratification in future clinical trials. The poorer PFS with first-line therapy suggests that *BRAF* mutation could be prognostic, predictive or both. As this association was highly significant for patients receiving oxaliplatin, irinotecan or both, as well as for patients receiving bevacizumab or not, the conservative interpretation is that *BRAF* mutation is a prognostic rather than a predictive marker.

Our observations have interesting implications for pathogenic mechanisms and disease subgroups. Activating mutations in both *KRAS* and *BRAF* stimulate the MEK or extracellular signal-regulated kinase–microtubule-associated protein (ERK–MAP) kinase cascade (Vogelstein and Kinzler, 2004). As the two mutations tend to be mutually exclusive (Rajagopalan *et al*, 2002; Yuen *et al*, 2002), *KRAS* is inferred to signal principally through *BRAF* in colon cancer cells, leading to an assumption that tumours with *KRAS* or *BRAF* mutations may be equally vulnerable to inhibition of *BRAF*. Our finding that the natural history and treatment response of *BRAF*-mutant tumours differ markedly from all others implies that *BRAF* mutation does not simply substitute for *KRAS* activation in a linear signalling pathway but probably confers additional or distinct properties, with ominous consequences. In cultured cells, the V600E mutation increases BRAF activity independent of *KRAS* and shows lower transforming activity than oncogenic KRAS (Davies *et al*, 2002). Likewise, small-molecule inhibition of MEK abrogated tumour growth more profoundly in *BRAF*-mutant than *KRAS*-mutant tumour xenografts (Solit *et al*, 2006). Such differences may in part underlie the contrast in predictive value of activating *KRAS* and *BRAF* mutations. Second, the frequency of *BRAF* mutation is especially high, up to 50%, in tumours with microsatellite instability (MSI^hi^) (Rajagopalan *et al*, 2002), which otherwise carry a favourable prognosis (Popat *et al*, 2005). If the outcome of patients with *BRAF* mutation is so poor, then the natural history of MSI^hi^ tumours lacking *BRAF* mutation must be even more favourable; a recent study of patients with operable stage II or stage III CRC made the same suggestion (French *et al*, 2008). If this idea is confirmed, patients with such tumors may, in the future, be candidates for prolonged observation without treatment.

In agreement with recent reports (Bokemeyer *et al*, 2008; Van Cutsem *et al*, 2009), mutant or WT status for *KRAS* did not correlate with the outcomes in patients treated with front-line 5FU-based regimens. Although a previous study reported worse outcomes in patients with early (stages II–III) *PIK3CA*-mutant CRC (Kato *et al*, 2007), the aggregate data do not currently suggest that *KRAS*- or *PIK3CA*-mutant tumours have distinctive clinical profiles or differential response to currently approved chemotherapy. The lack of overall predictive significance of *KRAS* mutation despite its effect on cetuximab treatment hints that the adverse effect of *BRAF* mutation is not explained just by the limited response to cetuximab.

This study reinforces the value of mutational analysis in patients treated with EGFR mAb. Patients with *KRAS*-mutant tumours treated with cetuximab in any combination had lower PFS compared with those with *KRAS*^WT^ tumours, results that extend those reported in recent clinical trials (Bokemeyer *et al*, 2008; Van Cutsem *et al*, 2009) and suggest that KRAS mutation may bypass aberrant EGFR signalling. We observed similar trends in the likelihood of response between patients with *BRAF*-mutant or *BRAF*^WT^ tumours (0 *vs* 17%). Although responses in *PIK3CA*-mutant cases were not clearly as worse as they were in cases with *KRAS* or *BRAF* mutation, PFS with cetuximab-based therapy was significantly lower when tumors carried mutations in any of the three examined genes, and both *BRAF* (HR 3.9, *P*=0.0005) and *PIK3CA* (HR 2.1, *P*=0.01) mutations conferred higher risk of relapse after treatment with cetuximab-containing salvage combinations. Two studies that previously considered the significance of *BRAF* and *PIK3CA* mutations in treatment response to anti-EGFR mAb (Moroni *et al*, 2005; Lievre *et al*, 2006) had small sample sizes (30 and 31 cases, fewer than three with mutations). Our study extends the current paradigm to imply that patients with *KRAS*-, *BRAF*- or *PIK3CA*-mutant tumours may all derive limited benefit from treatment with EGFR mAb, and that *BRAF* mutations in particular account for a measurable fraction of patients whose *KRAS*^*WT*^ tumours do not respond to cetuximab. Reduced PFS in *PIK3CA*-mutant mCRC is not simply a function of poorer prognosis because overall outcomes are unrelated to this genetic event (Figure 1). Indeed, *KRAS* and *PIK3CA* mutations were not associated with worse OS, possibly because 82% of patients received another regimen after progression on cetuximab-based treatment or because response to cetuximab impacts modestly on the natural history of mCRC. (Messersmith and Ahnen, 2008).

Colorectal tumours are typically accessible for molecular characterization and common mutations localize in specific exons. Although it is formally possible that the mutational profile of metastatic deposits differs from that in the primary tumour, recent analyses indicate that this is not the case, (Jones *et al*, 2008); in any event, our analysis shows certain molecular lesions in the primary tumor as markers of the clinical outcome in mCRC. As we studied a large number of unselected cases with good representation of index mutations, the results are robust and directly relevant to current management of mCRC. They advance application of CRC mutation profiles to define patient sub-groups and should influence patient selection or stratification for prospective trials. *KRAS* and *PIK3CA* mutations seem not to be useful predictors for response to current treatment regimens. If our conclusions are confirmed independently, then patients with the *BRAF* V600E mutation might justify foregoing approved treatments in favour of investigational therapy. It will also be instructive to confirm the idea that like *KRAS* mutations, those in *BRAF* and *PIK3CA* also predict for lack of clinical benefit from EGFR mAb therapy.

## Figures and Tables

**Table 1:** Treatment regimens used in this retrospective study

**First-line regimens**	**N (out of 168)**	**%**
FOLFOX+bevacizumab	76	45
FOLFIRI+bevacizumab	19	11
5FU+bevacizumab	2	1
FOLFOX+cetuximab	8	5
FOLFOXIRI	17	10
FOLFIRI	25	15
FOLFOX	16	10
5FU only	5	3
Oxaliplatin-based treatment (first line)	100	60
Irinotecan-based treatment (first line)	44	26
Bevacizumab+chemotherapy (first line)	97	58
Oxaliplatin-based treatment (any line)	144	86
Irinotecan-based treatment (any line)	121	72
Bevacizumab+chemotherapy (any line)	131	78
Cetuximab+chemotherapy (any line)	100	60
Patients treated with all three chemotherapy drugs	106	63
Patients treated with all five active agents	87	52

FOLFIRI=Folinic acid, 5FU, irinotecan; FOLFOX=Folinic acid, 5FU, oxaliplatin; FOLFOXIRI=Folinic acid, 5FU, oxaliplatin, irinotecan.

**Table 2:** Characteristics of enrolled patients and univariate analysis for survival

			**First-line progression-free survival**			**Overall survival**		
**Feature**	**N**	**%**	**Median (months)**	**HR (95% CI)**	***P*-value**	**Median (months)**	**HR (95% CI)**	***P*-value**
Median age (range)	59 (23–86)			1.1 (0.8–1.6)	0.5		1.7 (1.1–2.6)	0.03
⩽65 years	113	67	12.5			43.7		
>65 years	55	33	10.7			30.6		
								
*Gender*				1.1 (0.8–1.5)	0.8		1.0 (0.7–1.6)	0.8
Male	87	52	11.7			38.9		
Female	81	48	12.5			38.2		
								
*Stage at diagnosis*								
I–III	77	46	13.6	0.8 (0.6–1.1)	0.2	38.2	1.2 (0.8–1.8)	0.5
IV	91	54	11.6			35.2		
								
*Tumour location*								
Colon	135	80	11.3	1.8 (1.2–2.7)	0.008	33.5	2.0 (1.1–3.7)	0.02
Rectum	33	20	16.3			66.6		
Number of metastatic lesions	Median: 2							
1	50	30	20.5	0.4 (0.2–0.6)	<0.0001	56.8	0.4 (0.2–0.8)	0.008
2	83	49	11.7	0.8 (0.5–1.2)	0.2	34.8	0.8 (0.5–1.4)	0.4
⩾3	35	21	10.5			28.8		
								
*Number of treatment lines*	Median: 3							
1	43	25	44.5	0.3 (0.2–0.5)	<0.0001	35.2	1.6 (0.9–2.8)	0.1
2	47	28	11.5	0.9 (0.6–1.3)	0.7	34.8	1.4 (0.8)	0.2
⩾3	78	47	11.8			39.1		
								
*Histologic grade*				1.4 (1.0–2.1)	0.06		1.8 (1.1–2.8)	0.02
I–II	127	76	12.5			41.1		
III	41	24	9.4			33.7		
								
*Adjuvant treatment*				1.1 (0.8–1.6)	0.6		1.0 (0.7–1.6)	0.9
Yes	58	35	13.7			38.9		
No	110	65	11.7			39.1		
								
*Metastatectomy*				0.4(0.2–0.6)	<0.0001		0.6 (0.3–1.2)	0.1
Yes	30	18	27.4			46.3		
No	138	82	11.1			38.4		
								
*KRAS status*				0.9 (0.7–1.3)	0.7		1.1 (0.7–1.8)	0.6
Mutant	62	37	12.3			35.8		
WT	106	63	11.8			40.5		
								
*BRAF* status				4.9 (2.7–9.0)	<0.0001		4.5 (2.4–8.4)	<0.0001
Mutant	13	8	4.3			10.9		
WT	155	92	12.5			40.5		
								
*PIK3CA* *status*				1.3 (0.8–1.9)	0.3		1.0 (0.6–1.8)	0.99
Mutant	26	15	11.4			44.7		
WT	142	85	12.1			38.2		

HR=hazard ratio; WT=wild type.

**Table 3:** Results of multivariate analysis for progression-free and overall survival

	**Hazard ratio**	**95% CI**	***P*-value**
*Progression-free survival*			
*BRAF* (mutant *vs* WT^*^)	4.0	(2.2, 7.4)	<0.0001
Age (>65 *vs* ⩽65 years)	1.4	(1.0, 2.0)	0.06
Tumour grade (3 *vs* 1–2)	1.5	(1.0, 2.2)	0.06
Metastatectomy (yes *vs* no)	0.6	(0.4, 1.1)	0.08
Tumour location (colon *vs* rectum)	1.6	(1.0, 2.4)	0.05
Number of treatment lines (1 *vs* >3)	0.3	(0.2, 0.5)	<0.0001
Number of treatment lines (2 *vs* >3)	0.9	(0.6, 1.3)	0.5
			
*Overall survival*			
*BRAF* (mutant *vs* WT)	4.1	(2.1, 8.0)	<0.0001
Age (>65 *vs* ⩽65 years)	1.5	(1.0, 2.4)	0.07
Tumour grade (3 *vs* 1–2)	1.3	(0.8, 2.1)	0.3
Metastatectomy (yes *vs* no)	0.6	(0.3, 1.3)	0.2
Stage at diagnosis (I–III *vs* IV)	1.3	(0.8, 2.0)	0.2
Tumor location (colon *vs* rectum)	1.8	(0.9, 3.3)	0.08
Number of lines of treatment (1 *vs* ⩾3)	1.8	(0.98, 3.3)	0.06
Number of lines of treatment (2 *vs* ⩾3)	1.4	(0.8, 2.3)	0.3

CI=confidence interval; WT=wild type.
