# Supplementary material for: Prognostic and predictive value of common mutations for treatment response and survival in patients with metastatic colorectal cancer
Source: Br J Cancer. 2009 Jul 14;101(3):465–72. doi: 10.1038/sj.bjc.6605164 (PMC2720232; doi:10.1038/sj.bjc.6605164)
Supplement: Supplementary Tables 1–3 [file 6605164x1.doc]

				
		
	
			
					
			
			
	


		
		
		
		
		
		
		
		
		
		
		
		
		
		
		
		
		
		
		


		
		
		
		
		
		
		
		
		
		
		
		
		
		
		
		
		
		
		
		
					
							
							
								
								
								
								
								
								
								
								
								
								
								
						
								
								
								
						
								
								
								
								
								
								
								
								
								
								
								
								
								
								
								
								
								
								
								
								
								

		

		

					
					
					
					
					
					
					
					
					
					
					
					
					
					
					
				
				
				
				


		
		
			
			
			
		
		
		
		
		
		
		
		
		
		
		
		
		
		
		
		
		
		
		
		Supplemental Table 1. Primer sequences and conditions for PCR.
		
		
	MUTATIONS	PRIMERS	ANNEALING TEMPERATURE
KRAS Codon 12	G12A G12C G12D G12R G12S G12V G13D	5’-TTATAAGGCCTGCTGAAAATGACT-3’ 5’ TATCTGTATCAAAGAATGGTCCTGC-3’	59OC
BRAF	V600E D594K	5’-CTACTGTTTTCCTTTACTTACTACACCTCAGA-3’ 5’-ATCCAGACAACTGTTCAAACTGATG-3’	60OC
PIK3CA Exon 9	E542K E545K E545G E545D Q546K D549N	5’-TAAGGGAAAATGACAAAGAACAGCTCA-3’ 5’-TTAGCACTTACCTGTGACTCCATAGAAAATC-3’	62OC
PIK3CA Exon 20	R1023Q H1047R H1047L G1049A	5’-TCTTTTCTCAATGATGCTTGGC-3’ 5’-TGAAATACTCCAAAGCCTCTTGC-3’  5’-TAGCCTTAGATAAAACTGAGCAAGAGG-3’ 5’-ATGCATGCTGTTTAATTGTGTGG-3’	60OC   60OC

		
		
		
		

		Supplemental Table 2. Distribution of mutations between the two study sub-populations.
		
		
Gene locus	United States N=109 (%)	Greece N=59 (%)	Total N=168 (%)	p value
KRAS	43 (39)	19 (32)	62 (37)	0.3
BRAF	8 (7.5)	5 (8.5)	13 (8)	0.7
PIK3CA exon9	11 (10)	7 (12)	18 (11)	0.7
PIK3CA exon20	5 (4.5)	3 (5)	8 (5)	0.8

		

Suppl. Table 3. Association between specific mutations and chemotherapy outcomes.

	PFS^ (months)	HR^ (95% CI); p value	
All 1st line chemotherapy (N=168) Full cohort                                             12.0
KRAS Mutant (N=62)	12.3	0.9 (0.7-1.3); 0.69
WT (N=106)	11.8	
BRAF Mutant (N=13)	4.3	4.9 (2.7-9.0); <0.0001
WT (N=155)	12.5	
PIK3CA Mutant (N=26)	11.4	1.3  (0.8-1.9); 0.30
WT (N=142)	12.1	
Oxaliplatin-based first-line therapy (N=100) Full cohort                                                          11.7
KRAS Mutant (N=39)	11.7	1.1 (0.7-1.6); 0.82
WT (N=61)	11.4	
BRAF Mutant (N=6)	5.0	6.4 (2.6-15.6); <0.0001
WT (N=94)	11.7	
PIK3CA Mutant (N=19)	10.7	1.4 (0.8-2.4); 0.19
WT (N=81)	11.7	
Irinotecan-based first-line therapy (N=44) Full cohort                                                          12.2
KRAS Mutant (N=18)	13.7	0.5 (0.3-1.1); 0.074
WT (N=26)	12.0	
BRAF Mutant (N=5)	3.5	4.1 (1.5-11.3); 0.006
WT (N=39)	12.8	
PIK3CA Mutant (N=4)	13.0	1.0 (0.4-3.0); 0.94
WT (N=40)	12.2	
Bevacizumab-containing first-line therapy (N=97) Full cohort                                                          11.9
KRAS Mutant (N=39)	12.1	0.9 (0.6-1.5); 0.79
WT (N=58)	11.7	
BRAF Mutant (N=8)	4.2	5.1 (2.4-11.1); <0.0001
WT (N=89)	12.5	
PIK3CA Mutant (N=14)	12.0	1.1 (0.6-2.0); 0.71
WT (N=83)	11.9	
Cetuximab-containing salvage treatment (N=92) Full cohort                                                           3.8
KRAS Mutant (N=32)	2.5	1.5 (0.9-2.3); 0.094
WT (N=60)	4.8	
BRAF Mutant (N=9)	2.0	3.6 (1.8-7.4); 0.0005
WT (N=83)	3.9	
PIK3CA Mutant (N=13)	2.5	2.1 (1.2-3.9); 0.014
WT (N=79)	3.9	
Any Index Mutation (N=48)	2.5	2.1 (1.3-3.2); 0.001
WT at all 3 loci (N=44)	6.4	


 
